# Prioritisation of co-formulants and plant protection products for non-dietary risk assessment using NAMs

**DOI:** 10.1007/s00204-025-04078-0

**Published:** 2025-07-02

**Authors:** Alkiviadis Stagkos-Georgiadis, Bright Baffour-Duah, Tewes Tralau, Denise Bloch

**Affiliations:** 1 Department of Pesticides Safety, German Federal Institute for Risk Assessment (BfR), Max-Dohrn-Str. 8-10, 10589 Berlin, Germany; 2 Department of Nutritional Toxicology, Institute of Nutritional Science, University of Potsdam, Arthur-Scheunert-Alle 114-116, 14558 Nuthetal, Germany; 3 Department of Food Chemistry, Institute of Nutritional Science, University of Potsdam, Arthur-Scheunert-Alle 114-116, 14558 Nuthetal, Germany

**Keywords:** Co-formulants, NAMs, Pesticide risk assessment, Mixture effects, Kinetic interactions, Plant protection products, Agrochemical formulations

## Abstract

Plant protection products (PPPs) contain one or more active substances (AS) as well as a varying number of co-formulants. Whilst AS are amongst the most data-rich and well-investigated substances in toxicology, possibly toxic co-formulants and PPPs with potentially relevant mixture effects are not considered in risk assessment. This especially applies to operators who come in contact with the undiluted product. In this study, we investigated this concern by prioritising co-formulants and PPPs for further testing using new approach methodologies (NAMs). We combined in silico predictions of co-formulant nephrotoxicity and hepatotoxicity with AS and co-formulant kinetic interaction prediction and thus identified 427 PPPs for further investigation. In a next step, six PPPs were there assessed for their comparative AS and PPP toxicity in liver cells. One product displayed more than additive effects and was thus tested for in silico predicted kinetic interactions. Whilst permeability glycoprotein (P-gp) interaction could not be confirmed, the observed effect may likely be attributed to CYP2C19 interaction. In summary, the findings highlight that the use of in silico tools as a screening step is useful to limit the number of PPPs for further testing. Subsequent in vitro testing of a limited number of PPPs supported the application of the additivity concept and highlighted that prioritised co-formulants contribute additively. We propose the integration of such co-formulants into the Hazard Index (HI) approach currently applied in active substance combined risk assessment.

**Supplementary Information:**

The online version contains supplementary material available at 10.1007/s00204-025-04078-0.

## Introduction

Plant protection products (PPPs) are mixtures containing one or more active substances (AS) as well as several co-formulants. AS serve a functional role in PPPs, whereas co-formulants are important for their efficacy (Hazen 2000). The authorisation procedure for both AS and PPPs falls under Commission Regulation (EC) No 1107/2009 (EC 2009). In particular, AS hazard and risk are extensively evaluated prior to approval for different endpoints following Regulation (EU) No 283/2013 (EC 2013a), whilst PPP evaluation is limited to hazard assessment based on six acute and topological toxicity studies (“6 pack”) or NAM-based alternatives following Regulation (EU) No 284/2013 (EC 2013b). The impact of co-formulants on AS non-dietary risk assessment is currently limited to dermal absorption. In addition, co-formulants do not require any further toxicological evaluation or authorisation as part of PPP Regulation (EU) No 1107/2009 (EC 2009), but they are toxicologically assessed based on their annual production volume according to REACH Regulation (Regulation (EC) No 1907/2006) (EC 2006). Furthermore, a list of co-formulants not accepted for inclusion in PPPs is available as an amendment to Regulation (EC) No 1107/2009 ANNEX III (EC 2009). Notably, Regulation (EC) No 284/2013 on the data requirement for plant protection products states that, where relevant, the non-dietary exposure assessment shall “take into account cumulative and synergistic effects resulting from the exposure to more than one active substance and toxicologically relevant compounds, including those in the product and tank mix” (EC 2013b). However, in practice, only potentially additive effects of several active substances contained in the same PPP are considered in non-dietary risk assessment (EC 2008).

Mixture toxicity can be characterised by two approaches. If a mixture is fully chemically defined, the component-based approach is followed, whereas the whole mixture approach is applied when a mixture is poorly defined. The component-based approach most commonly applied in hazard and risk assessment is dose or concentration addition (CA) (More et al. 2019). Yet, this approach does not take into account the contribution of co-formulants in PPP toxicity and neglects kinetic interaction. Therefore, case-by-case assessment should be applied where synergistic effects are expected. However, current proposals for refinement are restricted to toxicodynamic mixture effects. The approach according to Stein et al. (2014) even suggests limiting CA to AS within the same cumulative assessment group (CAG), neglecting combined toxicity of substances across CAGs. Moreover, deviations from concentration/response addition models can also be attributed to toxicokinetic interactions between AS and co-formulants (Bloch et al. 2020).

In recent years, several in vitro studies concerning PPPs have been published, showing that products may exhibit increased toxic effects compared to the individual AS used (Hernandez et al. 2013; Zahn et al. 2018). Other studies have investigated mixture effects caused by the interaction of co-formulants and AS, indicating that co-formulants can cause differential toxicity when in combination with AS (Li et al. 2015; Karaca et al. 2021). In this context, liver and kidneys are considered two of the main sites of toxic action for pesticide-induced toxicity due to their relevance in toxicant transformation and toxicant excretion. Notably, in many cases, increased toxicity is suspected to be the consequence of toxicokinetic effects (Bloch et al. 2023).

Co-formulants may influence AS kinetics by increasing passive transport (Karaca et al. 2021), engaging in metabolic interaction (Lasch et al. 2021), or retarding AS excretion by inhibiting active efflux transporters (Gueniche et al. 2019). One of the scenarios to be considered when addressing toxicokinetic interactions on human transporters or metabolising enzymes in multi-compound mixtures is the concomitant presence of potential substrates and inhibitors of the same enzyme (Braeuning et al. 2022). This is crucial when absorption and metabolism are affected by other components of the pesticide mixture, potentially leading to increased toxicity. In fact, existing data suggest that pesticides can be either substrates or inhibitors for active efflux transporters (Chedik et al. 2018). In particular, in vitro and in vivo studies have shown that several non-ionic surfactants inhibit the permeability glycoprotein (P-gp) transporter which can lead to an increased absorption rate of drugs that are substrates of this transporter (Hanke et al. 2010; Cornaire et al. 2004). Furthermore, toxicokinetic interactions may occur when co-formulants significantly increase the uptake or absorption of the active ingredient leading to higher bioactivity or bioavailability (Kienzler et al. 2014). In addition, many CYP enzymes are involved in the metabolism of pesticides, leading to activation and/or detoxification reactions (Abass et al. 2012). In the case of co-formulants, it is known that surfactants inhibit CYP-mediated metabolism and thus, enhance the bioavailability of co-administered active substances (Cristiansen et al. 2011; Mountfield et al. 2000).

Interaction between co-formulants, active substances, or co-formulants and active substances are currently not considered in PPP risk assessment. New approach methodologies (NAMs), including non-animal models and computational tools, offer an effective way to assess potential mixture risks (Bopp et al. 2019). The prioritisation of mixtures of concern and the identification of risk drivers are crucial first steps in investigating whether operator risk is sufficiently addressed by current regulatory practices since operators are likely exposed to much higher pesticide concentrations (Bloch et al. 2020).

The aim of this work is to provide an efficient NAM-based testing regime to identify relevant PPPs, which may need to undergo refined non-dietary risk assessment. To this end, we used in silico prediction to identify liver and kidney toxic co-formulants. Moreover, we investigated whether kinetic interactions between AS and co-formulants lead to mixture effects relevant for PPP risk assessment.

## Materials and methods

### Co-formulants dataset

**Fig. 1:**
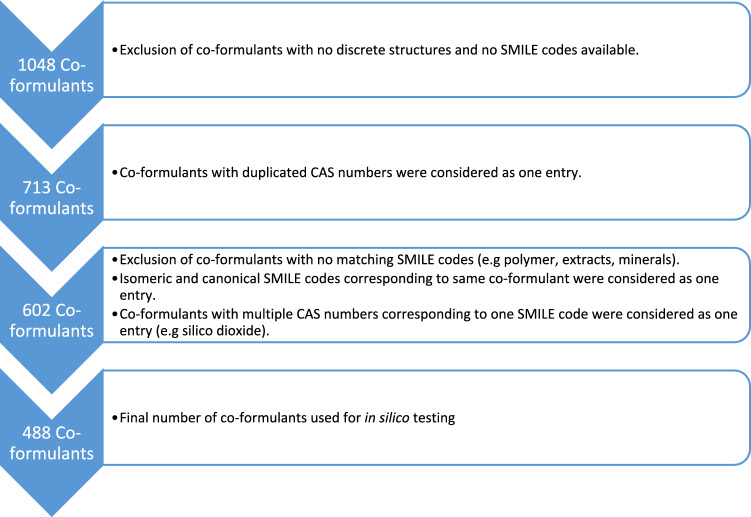
Extraction of co-formulants for further in silico testing

There are currently 1048 co-formulants used in the various authorised PPPs in Germany. Out of these, 488 were found to be unique when subsequently filtered for available simplified molecular-input line-entry systems (SMILES) and duplicated entries or substance classes. These were then used to investigate hepatotoxicity and nephrotoxicity endpoints using two Quantitative Structure–Activity Relationship (QSAR) software tools as well as potential absorption, distribution, metabolism, excretion ADME interactions (Fig. 1).

### In silico predictions for hepatotoxicity and nephrotoxicity using two (Q)SAR software tools

In general, in silico tools are divided into ruled-based models and statistical models. The combination of both models is recommended in order to reduce the number of false-positive and false-negative predictions (Benigni et al. 2019). In line with this approach, the rule-based model Derek Nexus (Lhasa Limited, version 6.2.1) for the endpoints hepatotoxicity and nephrotoxicity (i.e. sub-models (a) alpha-2-mu globulin nephropathy, (b) kidney disorders, (c) kidney function-related toxicity and (d) nephrotoxicity) was selected for mammalian species. In particular, Derek Nexus assessed the predictions of the aforementioned endpoints based on the literature references, evaluating alerts and estimating the likelihood of occurrence. The rules can be based on alerts as well as other types of evidence including physicochemical properties and known toxicological data (Marchant et al. 2008). The compounds were considered to have a structural alert for the selected endpoints if the prediction was “certain”, “probable”, “plausible” or “equivocal”. Predictions referred as “doubted”, “improbable”, “impossible”, “inactive” or “no alert” were regarded as negative. When Derek Nexus has no knowledge on which to base a prediction, the message “nothing to report” is displayed. All *likelihood levels* above equivocal (i.e. “plausible”, “probable”, “certain”) are generally accepted by regulatory authorities and included in the toxicological evaluation. Tautomers and mixtures were also included in the predictions.

In addition, the statistical model Leadscope Model Applier (Leadscope Inc., version 3.1) was selected for the following hepatotoxic endpoints: (a) bile duct disorder, (b) cholestasis, (c) liver damage and (d) liver enzyme abnormality, whereas the endpoints: (a) bladder disorder, (b) blood in urine, (c) kidney functional test abnormality, (d) nephropathy, (e) renal disorder and (f) urolithiasis were considered for the nephrotoxicity endpoint. In both cases, predictions with a positive alert were accepted. In particular, both the hepatotoxic and nephrotoxic suite of models follow the binary principle (0, 1), where the positive effects are trained as binary 1 and the negative effects as binary 0 based on a set of different descriptors. The outcome of the (Q)SAR prediction is given as the probability of being a toxicant on a scale of 0 to 1. The lower the probability value, the lower the potential toxicity of a test chemical is. The level of concern for the test chemicals’ toxicant potential is evaluated in proportion to the number of sets of models with positive predictions. Moreover, Leadscope Model Applier uses two parameters to guide the applicability of the model domain: (1) having at least one structural feature defined in the model in addition to all the property descriptors; (2) having at least one chemical in a training neighbourhood with at least 30% global similarity to the test structure. The training dataset for both endpoints as well as their respective sensitivity and specificity is provided in supplementary material (SM1).

### In silico kinetic predictions using ADMET predictor

In general, the commercial software tool ADMET Predictor (Simulations Plus) is constructed based on machine learning, and it can predict drug properties from multiple perspectives, including absorption and metabolism. ADMET Predictor models have a defined applicability domain in the interval (0, 1), and thus, any compound with descriptor values falling below −0.1 or above 1.1 in the training set range is marked as “out of scope,” indicating that it is out of the model’s applicability domain. Predictions are indicated with confidence; “NO” is provided for “out of scope” predictions, whereas predictions with confidence in brackets indicate “in scope” predictions. In our case, ADMET Predictor version 11 was used to predict certain ADME properties for both co-formulants and AS. The list of 185 hepatotoxic AS is selected from the CAGs (Nielsen et al. 2012). The list of 488 co-formulants and 185 hepatotoxic AS with their respective SMILES converted to Structure-data format (SDF) file, and a final number of 485 co-formulants and 183 hepatotoxic AS were attributed to a prediction value (SM2). In particular, the list of 485 co-formulants was used to assess potential inhibitors of the five main CYP450 isoenzymes (1A2, 2C9, 2C19, 2D6 and 3A4) as well as an active efflux transporter (P-gp), whereas a list of 183 AS responsible for hepatotoxic endpoints was used to test for potential substrates for the same targets. For both substrates and inhibitors, predictions with confidence above 50% were accepted. The training dataset for the endpoints investigated as well as their respective sensitivity and specificity is reported in the ADMET Predictor software manual (version 11.0, June 2023).

### Total sales of co-formulants based on annual production volume of PPPs

The list of 1048 co-formulants applied in different authorised PPPs in Germany was used to calculate their total sales taking into account PPP sales according to Eq. (1). Co-formulants with the same CAS numbers were considered as one individual entry, and their respective sales were summed up resulting in a total number of 853 co-formulants:

1 $${\text{Co - formulant sales }}\left( {{\text{kg}}} \right) = {\text{PPP sales }}\left( {{\text{kg}}} \right) \times {\text{co - formulant in PPP}} \, \left( \% \right)$$

### Test compounds

With respect to AS, cypermethrin (Cyp) (CAS no. 52315-07-8; batch no. BCCB1117; purity 99%), pendimethalin (Pen) (CAS no. 40487-42-1; batch no. BCCJ7317; purity 98.7%), flufenacet (Fluf) (CAS no. 142459-58-3; batch no. BCCF6599; purity 99.1%), pinoxaden (Pin) (CAS no. 243973-20-8; batch no. BCCJ8265; purity 99.1%), cloquintocet-mexyl (Clo) (CAS no. 99607-70-2; batch no. BCCF4868; purity 99.4%), deltamethrin (Del) (CAS no. 52918-63-5; batch no. BCCF1130; purity 98.6%), prothioconazole (Pro) (CAS no. 178928-70-6; batch no. BCCB2271; purity 99.9%) and tebuconazole (Teb) (CAS no. 107534-96-3; batch no. BCCH6660; purity >99.6%) were purchased from Sigma-Aldrich (Taufkirchen, Germany). Referring to co-formulants, 1-butylpyrrolidin-2-one (BP) (CAS no. 3470-98-2; batch no. B02828196) and N,N-dimethyldecanamide (NDA) (CAS no. 14433-76-2; batch no. B02827729) were purchased from Sigma-Aldrich (Taufkirchen, Germany), whereas solvent naphtha (SN) (CAS no. 64742-94-5; batch no. 209469; purity >98%) and 1-Octyl-2-pyrrolidone (NOP) (CAS no. 2687-94-7; batch no. 233356; purity >99%) were obtained from Molekula Group (Darlington, UK). The products were purchased from the manufacturer. All active substances, co-formulants and PPPs were dissolved in dimethyl sulfoxide (DMSO; CAS no. 67–68-5; batch no. K55014650314), obtained from Sigma-Aldrich (Taufkirchen, Germany), resulting in a final DMSO concentration of 0.5% (v/v) in treatment medium. One exception is the co-formulant tris-(2-ethylhexyl)-phosphate (TP) (CAS no. 78-42-2; batch no. BCCK1448; purity >99%) which was dissolved in (acetonitrile; CAS no. 75-05-8; batch no. I1223729), purchased from Merck (Darmstadt, Germany) resulting in a final acetonitrile concentration of 0.5% (v/v) in treatment medium.

### Cell culture

HepaRG cells were obtained from Biopredic International (Saint Grégoire, France) and were cultured for 2 weeks in Williams medium (Pan-Biotech GmbH, Aidenbach, Germany) containing 10% foetal calf serum (FCS Good Forte EU approved; PAN Biotech GmbH, Aidenbach, Germany), 100 U/ml penicillin, 100 μg/ml streptomycin (Capricorn Scientific GmbH, Ebsdorfergrund, Germany), 0.05% human insulin (Pan-Biotech GmbH, Aidenbach, Germany) and 50 μM hydrocortisone hemisuccinate (Sigma-Aldrich, Taufkirchen, Germany). Afterwards, cells were differentiated for an additional 2 weeks in differentiation medium. This medium is similar to the proliferation medium but supplemented with 1.7% DMSO. After differentiation and 24 h before experiments, cells were transferred to treatment medium. The latter corresponds to the differentiation medium but contains 2% FCS and 0.5% DMSO only.

HepaRG cells were incubated at 37 °C in a 5% CO_2_, 5% humidity atmosphere in a Binder cell culture incubator. Cells were passaged every week until passage five. Passaging was performed by aspirating the proliferation medium followed by subsequent washing of the cells with phosphate-buffered saline (PBS) and incubation in Dulbecco’s Phosphate-Buffered Saline (Capricorn Scientific GmbH, Ebsdorfergrund, Germany) supplemented with 2 ml trypsin-EDTA (0.05%) at 37 °C for 2 min. Trypsinization was stopped by adding proliferation medium. Cells were then seeded into 96-well plates at a density of 9×10^3^ cells per well.

### Cytotoxicity assays

Cytotoxicity was first analysed by water soluble tetrazolium assay (WST-1, Roche, Berlin, Germany) followed by neutral red uptake (NRU). Cytotoxicity assays were performed in 96-well plates at a density of 9 × 10^3^ cells per well, and the cells were treated with the active substances, all the above-mentioned co-formulants individually, the combination of the active substances, the combinations of both active substances and co-formulants as well as the formulations (eight different concentrations in treatment medium with a final solvent concentration of 0.5% DMSO) for 24 h. The detergent Triton X-100 (0.01%) served as positive control. After 24-h incubation, 10 μl of WST-1 was added to each well according to the protocol provided by Roche. After 30 min of incubation with WST-1, absorbance was measured (*λ*_exc_ = 450 nm/*λ*_emi_ = 620 nm) on an Infinite M200 PRO plate reader (Tecan, Männedorf, Swiss). In addition, NRU assay was performed according to the protocol by Repetto et al. (2008). In brief, wells containing medium with WST-1 were removed, and cells were washed with 100 μl PBS per well. Subsequently, 100 μl of neutral red medium was added per well, and cells were incubated for 2 h at 37 °C. Afterwards, neutral red medium was removed, the wells were washed with 100 μl PBS, and 150 μl of a solution (50% ethanol/49% Milli-Q water/1% acetic acid) was added per well to extract the neutral red dye. The plates were shaken for 10 min at room temperature, and therefore, the fluorescence of the absorbed dye was measured (*λ*_exc_ = 530 nm/*λ*_emi_ = 645 nm) on an Infinite M200 PRO plate reader (Tecan, Männedorf, Swiss). Background-corrected viability values of both assays were expressed as a percentage of untreated cells. Three individual biological replicates were performed, and each concentration was measured in three technical replicates. Means and standard deviations were calculated.

### Concentration addition modelling

Possible deviations from dose addition and the occurrence of synergistic or antagonistic effects were analysed by dose–response modelling in PROAST software (v. 70.1, https://proastweb.rivm.nl/, RIVM, Bilthoven, The Netherlands) taking into account the cytotoxic effects of the investigated AS and co-formulants. The highest concentration tested for all treatments except two co-formulants was approximately 300 mg/L and higher concentrations than that were not considered relevant. Dose–response curves of the combinations of AS and co-formulants were used to estimate a theoretical mixture curve under the assumption of dose addition. As described in Kienhuis et al. 2015, we fitted a four parameter exponential model: *y* = *a* * [*c* − (*c* − 1) * exp(−(*x/b*)^*d*)] to the substances Pro, NOP, BP (Product 4), Pen+Fluf, SN (Product 5) and Pro+Teb, NDA (Product 6). PROAST consecutively shows the analysis based on the Exponential and Hill models, and thus the best fitted models were used depending on the lowest Akaike Information Criteria (AIC). In addition, dose–response data of the mixtures Pro+NOP+BP, Pen+Fluf+SN and Pro+Teb+NDA were plotted in addition to the single dose–response data, and therefore, the mixtures were compared with the respective theoretical mixture curves under the assumption of concentration addition (Kienhuis et al. 2015; Lasch et al. 2020). Concentration addition can be assumed, if the data points of the AS, co-formulants and their respective combinations fit with the theoretical mixture curves. A more than additive/synergistic effect can be assumed if data points are left shifted compared with the theoretical mixture curves, whereas a less than additive/antagonistic effect can be assumed when data points are right shifted compared with the theoretical mixture curves.

### P-gp ATPase assay

To evaluate the substrate and inhibition relationship of Pro and NDA with P-gp, respectively, SB-MDR1/P-gp PREDEASY ATPase kit (Solvo Biotechnology, Budaörs, Hungary) was used following the protocol provided by the manufacturer. ATPase activity in the presence of Pro and NDA was estimated by measuring the release of inorganic phosphate (P_i_) in a colorimetric reaction over a concentration range of 0.05–103 mg/L and 0.03–59.8 mg/L, respectively. Both substances were dissolved in DMSO (2% final concentration).

In brief, MDR1-Sf9 membrane vesicles (4 μg/well) were incubated in 10 μl MgATP solution (2 mM) with the test substances for 10 min at 37 °C using two distinct protocols:

- Activation study: incubation of Pro with or without 1.2 mM sodium orthovanadate (Na3VO4).
- Inhibition study: incubation of NDA simultaneously with 40 μM (18.2 mg/L) verapamil (a known P-gp substrate used in this assay as the reference P-gp activator) and with or without 1.2 mM Na3VO4 incubation.

The reaction was stopped by adding 100 µL of Developer solution to each well, followed by 100 µL of Blocker solution and an additional 30 min incubation at 37 °C. The absorbance was measured at 620 nm using an Infinite M200 PRO plate reader (Tecan, Männedorf, Swiss) reflecting the amount of inorganic phosphate (*P*_i_) liberated by the transporter which is proportional to its ATPase activity. The MDR1-Sf9 membrane vesicles contain other ATPases besides P-gp. As P-gp is effectively inhibited by Na3VO4, P-gp ATPase activity was measured as the vanadate-sensitive portion of the total ATPase activity. Thus, ATPase activities were determined as the difference of *P*_i_ liberation measured in the absence or presence of 1.2 mM sodium orthovanadate (i.e. vanadate-sensitive ATPase activity) and expressed as nmol *P*_i_ liberated/mg protein/min. The amount of *P*_i_ liberation was calculated from a phosphate standard curve. Cyclosporine A (40 µM) (48.1 mg/L) was used in the present assay as the positive control for the inhibition studies, and the corresponding activity was referred to as inhibited ATPase activity. Compounds were evaluated in duplicates, and the assay was performed twice. The results were expressed as vanadate-sensitive ATPase activities.

### P450-Glo™ CYP1A2 and CYP2C19 inhibition assay

The assays focussed on potential inhibitory effects on human cytochrome P450 CYP2C19 and CYP1A2 enzyme activity. This inhibition study relied on recombinant human CYP2C19 and CYP1A2 enzymes integrated in membrane preparation since additional metabolising enzymes present in HepaRG cell lines may lead to potential cross-reactivity. For both P450-Glo™ CYP2C19 and CYP1A2 Screening Systems obtained from Promega (Cat.#V8882, Cat.#V4880, Cat.#V8772, Cat.#V4770, Madison, Wisconsin, USA), negative control membranes, Luciferin-H EGE (CYP2C19) and Luciferin-ME (CYP1A2) as luminogenic substrate, NADPH regeneration system, Potassium Phosphate Buffer, Luciferin Detection Reagent and Luciferin-Free Water were provided. NDA was tested up to 1.99 mg/L for CYP2C19 enzyme inhibition, and SN was investigated up to 150 mg/L for CYP1A2 enzyme inhibition in eight different concentrations. Both substances were dissolved in DMSO resulting in a final concentration of 0.4% (v/v) in water. White 96-well plates were used, and the assay was conducted following the manufacturer's user protocol (Promega, Madison, Wisconsin, USA).

With respect to CYP2C19 assay, 0.25 μl recombinant CYP2C19 membranes, 0.05 μl Luciferin-H EGE (10 mM) and 2.5 μl Potassium Phosphate Buffer (1 M) in Luciferin-Free Water were incubated with 12.5 μl of eight different concentrations of the test compound. Furthermore, CYP2C19 membranes were incubated with 12.5 μl troglitazone (final concentration 10 μM, 4.42 mg/L) as CYP2C19 inhibition control or 12.5 μl water (final concentration 0.4% DMSO) as vehicle control. Minus P450 control wells were incubated only with control membranes. Plates were shaken on a plate shaker and preincubated for 10 min at room temperature. In addition, 25 μl of NADPH regeneration system (containing 2.6 mM NADP+, 6.6 mM glucose-6-phosphate, 6.6 mM MgCl_2_ and 0.8 U/ml glucose-6-phosphate dehydrogenase) was added to each well and plates were incubated for 30 min at room temperature to initiate the reaction. Luminescence of the luciferin product was initiated by adding 50 μl of Luciferin Detection Reagent. After briefly shaking on a plate shaker, plates were incubated for 20 min at room temperature in order to stabilise the luminescent signal.

Regarding CYP1A2 assay, 0.5 μl recombinant CYP1A2 membranes, 1 μl Luciferin-ME (5 mM) and 5 μl potassium phosphate buffer (1 M) in Luciferin-Free Water were incubated with 12.5 μl of eight different concentrations of the test compound. Moreover, CYP1A2 membranes were incubated with 12.5 μl α-naphthoflavone (final concentration 1 μM, 0.272 mg/L) as CYP1A2 inhibition control or 12.5 μl water (final concentration 0.4% DMSO) as vehicle control. Minus P450 control wells were incubated only with control membranes. Plates were shaken on a plate shaker and preincubated for 10 min at 37 °C. In addition, 25 μl of NADPH regeneration system (containing 2.6 mM NADP+, 6.6 mM glucose-6-phosphate, 6.6 mM MgCl_2_ and 0.8 U/ml glucose-6-phosphate dehydrogenase) was added to each well and plates were incubated for 10 min at 37 °C to initiate the reaction. Luminescence of the luciferin product was initiated by adding 50 μl of Luciferin detection reagent. After briefly shaking on a plate shaker, plates were incubated for 20 min at room temperature in order to stabilise the luminescent signal.

Luminescence signals were measured on the Infinite M200 PRO plate reader (Tecan, Männedorf, Swiss), and all the measurements were corrected by subtraction of minus P450 control signals (non-CYP2C19 and CYP1A2 activity). Basal CYP1A2 and CYP2C19 activities are represented by the difference in luminescence signals of minus P450 controls and untreated controls. ΔRLU values of the test compounds were compared to ΔRLU values of untreated samples (basal values) and represented as relative changes in luminescence signals. Each concentration was measured in three replicates (*n* = 3) and an IC_50_ value was calculated for NDA.

### Statistical analysis

Statistical analysis, graphical visualisation, EC_50_/IC_50_ calculation and curve fitting of the data were performed using Graphpad Prism software (Version 10.1.2). For cytotoxicity, P-gp ATPase assay, CYP2C19 and CYP1A2 inhibition assays, a linear mixed-effects ANOVA (*α* = 0.05) described by Pinheiro and Bates (2000) followed by a post hoc Dunnett test of multiple comparisons of treatment groups vs. the control, was used for statistical analysis.

## Results

### Scoring criteria for positive predictions obtained by Derek Nexus and Leadscope Model Applier

**Fig. 2:**
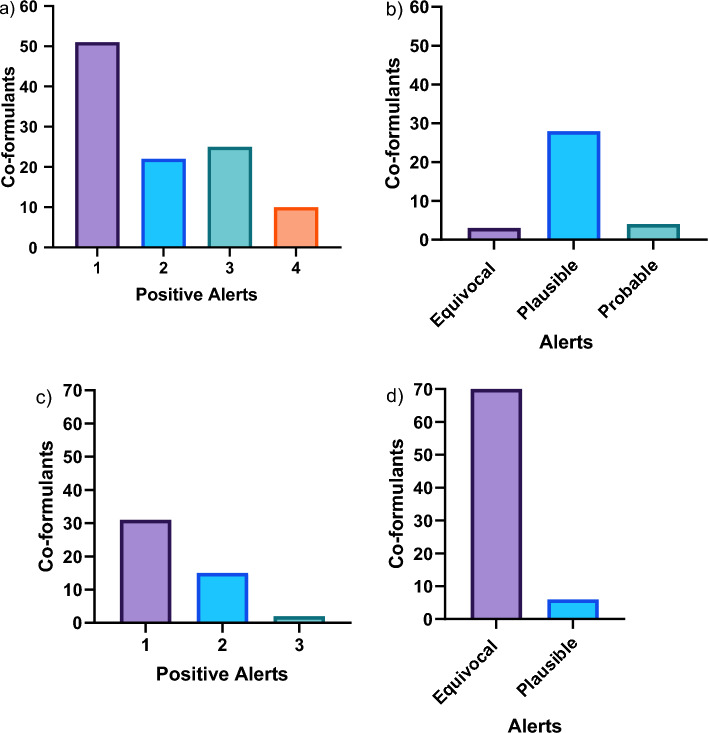
Number of co-formulants with positive alerts using Leadscope Model Applier statistical model and Derek Nexus expert model for **a, b** hepatotoxicity and **c, d** nephrotoxicity endpoints

**Fig. 3:**
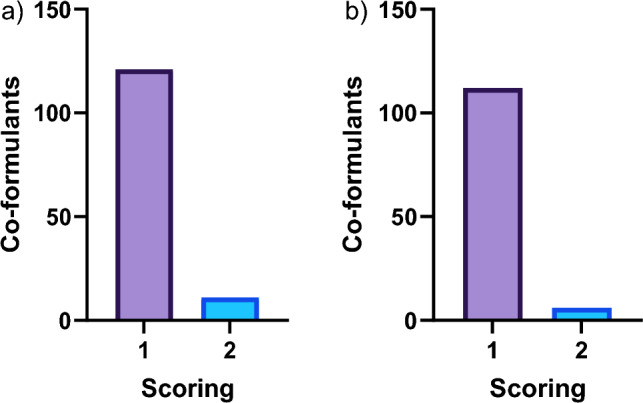
Number of co-formulants subjected to scoring for different **a** hepatotoxic and **b** nephrotoxic endpoints

**Fig. 4:**
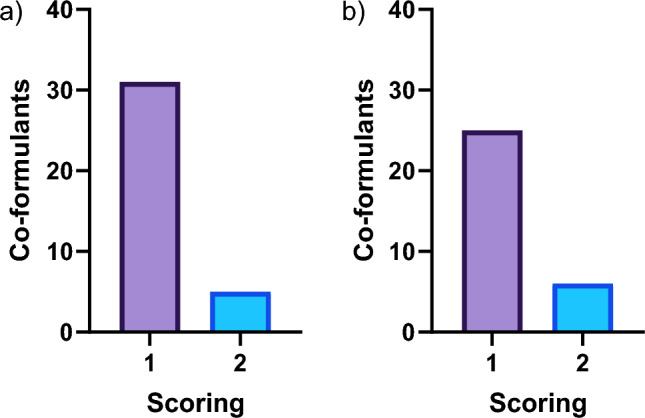
Number of **a** hepatotoxic and **b** nephrotoxic co-formulants with a score of 1 and 2 exceeding 10% and 0.1% in PPPs, respectively

With regard to hepatotoxicity, the statistical model (i.e. Leadscope Model Applier) flagged 51 co-formulants with one positive alert, 22 co-formulants with two positive alerts, 25 co-formulants with three positive alerts and 10 co-formulants with four positive alerts out of the total four hepatotoxic models. In addition, the expert-based model (i.e. Derek Nexus) flagged three co-formulants as “equivocal”, 28 co-formulants as “plausible*”* and four co-formulants as “probable” (Fig. 2a, b). A criterion covering the two software tools was created in order to rank the co-formulants with positive predictions. With respect to Leadscope Model Applier, a score of 1 was set if at least 1 positive alert was triggered. Concerning Derek Nexus, the predictions with a likelihood level of at least “equivocal” were given a score of 1. The scores 1 and 2 were given priority for hepatotoxicity, and thus, eleven co-formulants received a score of 2 and 121 a score of 1 (Fig. 3a). PPPs containing these co-formulants were prioritised for further testing if their percentage in the formulated PPP exceeded 0.1% (score of 2) or 10% (score of 1), respectively. Notably, five co-formulants with a score of 2 exceeded 0.1% in 21 PPPs, whilst 31 co-formulants with a score of 1 exceeded 10% in 143 PPPs (Fig. 4a).

Referring to nephrotoxicity, the statistical model (i.e. Leadscope Model Applier) flagged 31 co-formulants with one positive alert, 15 co-formulants with two positive alerts and two co-formulants with three positive alerts out of the total six nephrotoxic models. In addition, the expert-based model (i.e. Derek Nexus) flagged 70 co-formulants as “equivocal” whereas seven co-formulants flagged as “plausible” (Fig. 2c, d). The same aforementioned criteria considering the two software tools were also applied for the nephrotoxicity endpoint, and therefore, six co-formulants received a score of 2 and 112 a score of 1 (Fig. 3b). Notably, six co-formulants with a score of 2 exceeded 0.1% in 74 PPPs, whereas 25 co-formulants with a score of 1 exceeded 10% in 166 PPPs (Fig. 4b).

Hepatotoxic and nephrotoxic alerts are provided in as supplementary material, along with their respective scoring (SM3, SM4).

### In silico kinetic predictions using ADMET predictor

**Fig. 5:**
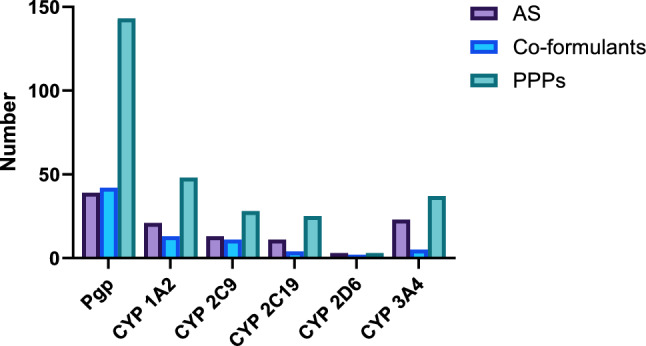
Columns indicate the number of co-formulants in authorised PPPs and their respective hepatotoxic AS. AS are selected from CAGs. For each endpoint, co-formulants are investigated as potential inhibitors (confidence >50%), whereas AS are investigated as potential substrates (confidence >50%) using ADMET Predictor

Based on ADME predictions, a total number of 143 PPPs were prioritised on which 42 co-formulants were flagged as a potential inhibitors and concomitantly 39 hepatotoxic AS as potential substrates for the P-gp efflux transporter. Concerning CYP1A2, 48 PPPs were prioritised on which 13 co-formulants were flagged as a potential inhibitors and 21 hepatotoxic AS as a potential substrates. In addition, 28 PPPs were prioritised with respect to CYP2C9, of which 11 co-formulants were identified as possible inhibitors and 13 hepatotoxic AS as possible substrates. Regarding CYP2C19, 25 PPPs were prioritised on which four co-formulants were flagged as potential inhibitors and 11 hepatotoxic AS as potential substrates. Based on CYP2D6, only three PPPs were given priority, out of which two co-formulants were identified as possible inhibitors and three hepatotoxic AS as possible substrates. Moreover, CYP3A4 was the focus of 37 PPPs, of which five co-formulants were identified as potential inhibitors and 23 hepatotoxic AS as possible substrates (Fig. 5). In all cases, co-formulants exceeded 0.1% in the formulated PPPs.

### Total sales of co-formulants in Germany

In order to filter for the 10 most relevant co-formulants, amounts were estimated based on their respective sales numbers from 2021. Table 1 contains the list of the top 10 “best-selling” co-formulants, with the complete list of 852 co-formulants being accessible in supplementary material (SM5).

**Table 1:** List of top 10 “best-selling” co-formulants contained in different PPPs in Germany 2021

CAS number	Co-formulants name	Co-formulants sales in tonnes
7732-18-5	Water	22962
130498-22-5	Wheat flour	3580
14433-76-2	N,N-Dimethyldecan-1-amide	3160
64742-94-5	Solvent naphtha (petroleum), heavy arom. (C9-C16)	1819
1189173-42-9	Hydrocarbons, C10, aromatics, <1% naphthalene	1222
57-55-6	Propane-1,2-diol	1078
61791-12-6	Castor oil, ethoxylated	974
85586-25-0	Fatty acids, rape-oil, Me esters	787
186817-80-1	Propanoic acid, 2-hydroxy-, 2-ethylhexyl ester, (2S)-	723
7783-20-2	Ammonium sulphate	508

### Selection of PPPs for further in vitro testing

Regarding hepatotoxicity, Product 1 was selected for further in vitro cytotoxicity testing along with its respective AS since a co-formulant (i.e. 1-(4-methyl-2-nitrophenylazo)−2-naphthol) with a score of 2 was evident in its content. Notably, the co-formulant triggered a positive alert in all statistical models and a “plausible” alert according to the expert-based model.

Relating to a score of 1, TP flagged as a “plausible” alert for hepatotoxicity endpoint (expert-based model) and Pip triggered a positive alert for “liver enzyme abnormality” (statistical model), and thus, both of them were selected for further in vitro cytotoxicity testing along with their respective PPPs and AS (i.e. Product 2 and 3). The latter co-formulant was also flagged as a potential inhibitor of CYP3A4, whereas Del was flagged as a potential CYP3A4 substrate according to ADMET Predictor. However, Pip is a synergist according to Regulation (EU) No 1107/2009, and thus, we did not follow up investigating kinetic interactions in vitro.

In addition, Product 4 was prioritised since BP triggered a positive alert for “liver enzyme abnormality” (statistical model) and concomitantly NOP flagged as a potential inhibitor of P-gp, whereas Pro flagged as a potential P-gp substrate according to ADMET Predictor.

Furthermore, Product 5 was prioritised for further in vitro testing since SN is the 4^th^ “best-selling” co-formulant according to our calculations.

Lastly, Product 6 was chosen for further testing since NDA was flagged as a potential CYP2C19 and P-gp inhibitor, whereas Teb and Pro were flagged as potential substrates of CYP2C19 and P-gp, respectively. In addition, NDA was listed as the 3^rd^ “best-selling” co-formulant according to our calculations.

### Cell viability

**Fig. 6:**
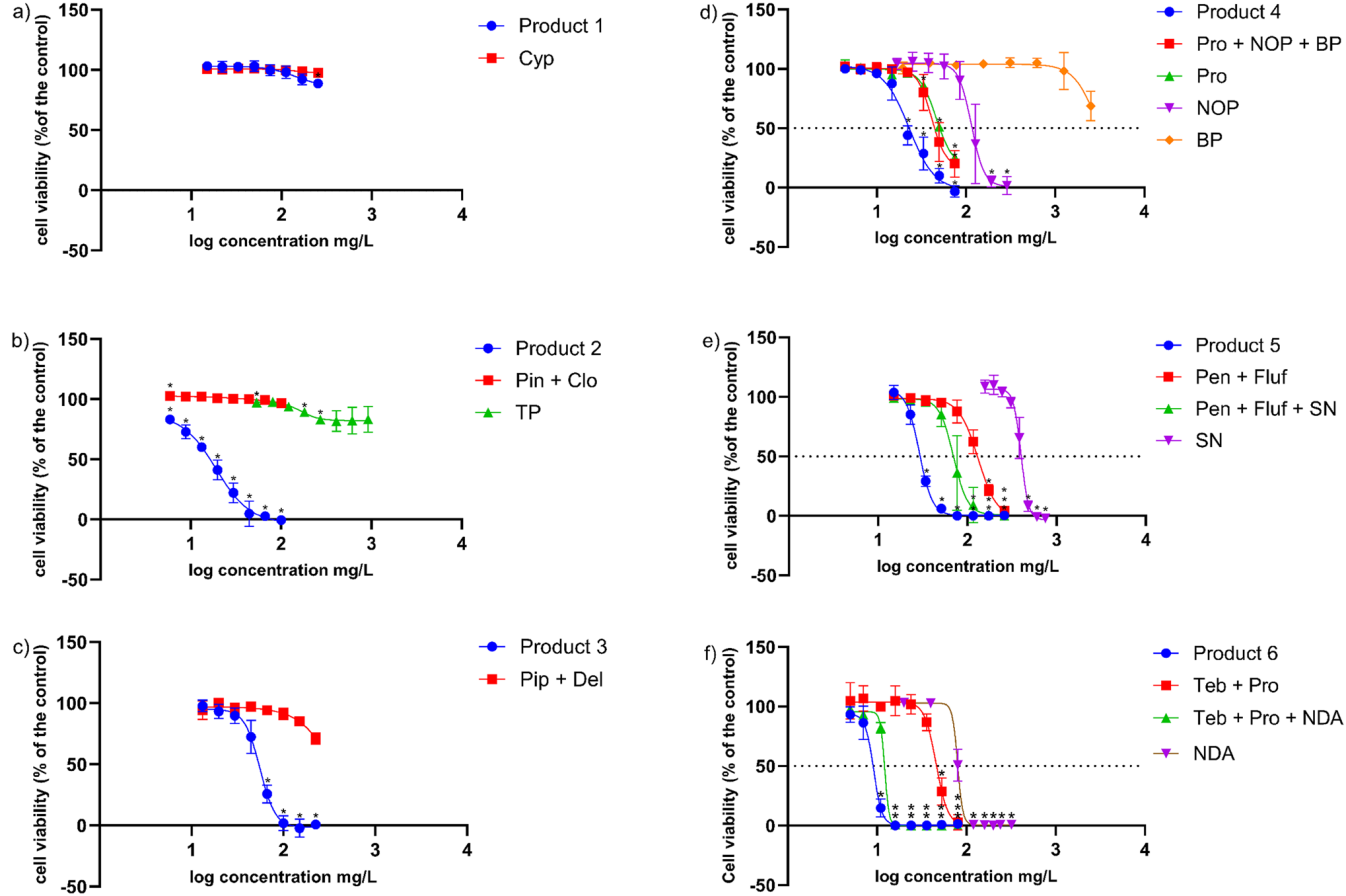
Results of the NRU cytotoxicity assay on HepaRG cells after 24-h exposure to increasing concentrations of **a** Product 1 and Cyp as featured in Product 1; **b** Product 2, a combination of Pin + Clo as featured in Product 2 and TP; **c** Product 3, a combination of Pip + Del and Pip as featured in Product 3; **d** Product 4, a combination of Pro + NOP + BP and Pro as featured in Product 4, NOP and BP; **e** Product 5, a combination of Pen+Fluf and a combination of Pep + Fluf + SN as featured in Product 5 and SN; **f** Product 6, a combination of Teb + Pro and a combination of Teb + Pro + NDA as featured in Product 6 and NDA. Mean values SD of *n* = 3 biological replicates each performed with three technical replicates. Statistical analysis was performed by a linear-mixed-effects ANOVA (*α* = 0.05) followed by a post hoc Dunnett test. Statistical significance compared to solvent control is indicated by an asterisk (*)

Cytotoxicity assays were used as a pre-screening test to prioritise PPPs which dominate AS cytotoxicity taking into account the potential cytotoxic contribution of co-formulants when added to the combination of AS, where needed. The overall cytotoxicity of all the test treatments was analysed by NRU assay after 24 h of incubation with HepaRG cells (Fig. 6).

Concerning Product 1, neither Cyp nor the product exhibited cytotoxic effects up to the highest concentration tested (Fig. 6a). In contrast, Product 2 was more cytotoxic compared to its AS; however, the difference was not caused by TP. TP’s in silico prediction was not confirmed in HepaRG cells (Fig. 6b). In the matter of Product 3, the combination of the two active substances was less cytotoxic compared to Product 3 for the same concentrations tested indicating that, despite the in silico predictions, pip did not contributed to the overall cytotoxicity of the product (Fig. 6c). Taking into account Product 4, both NOP and BP were flagged according to the in silico predictions but when in mixture with pro, the overall cytotoxicity of the mixture did not reflect the cytotoxicity occurred in Product 4 (Fig. 6d). In terms of Product 5, SN was investigated for its potential to contribute in the overall cytotoxicity when combined with Pen and Fluf. In fact, the mixture was more cytotoxic in comparison to the combination of Pep and Fluf but still less cytotoxic when compared to Product 5 (Fig. 6e). With respect to Product 6, NDA drives the cytotoxicity when added in the mixture of Teb and Pro resulting in an overall cytotoxicity matching the one encountered in Product 6 and thus, NDA’s in silico prediction is confirmed in HepaRG cells (Fig. 6f).

The results were also confirmed using WST-1 assay which showed mostly similar results (SM6). In addition, EC_50_ values were derived for Products 4, 5 and 6 as well as for their individual AS, co-formulants and combinations (Table 2).

**Table 2:** EC_50_ values based on cytotoxicity derived from best fit values of a non-linear dose–response curve of treatment substances on HepaRG cells after 24-h incubation

Test substances	EC_50_ (mg/L)
Product 4	22.97
Pro + NOP + BP	41.11
Pro	46.31
NOP	114.9
BP	1427
Product 5	28.99
Pen + Fluf + SN	69.98
Pen + Fluf	131.7
SN	399.4
Product 6	9.14
Teb + Pro + NDA	12.16
Teb + Pro	45.84
NDA	79.8

### Dose addition modelling

**Fig. 7:**
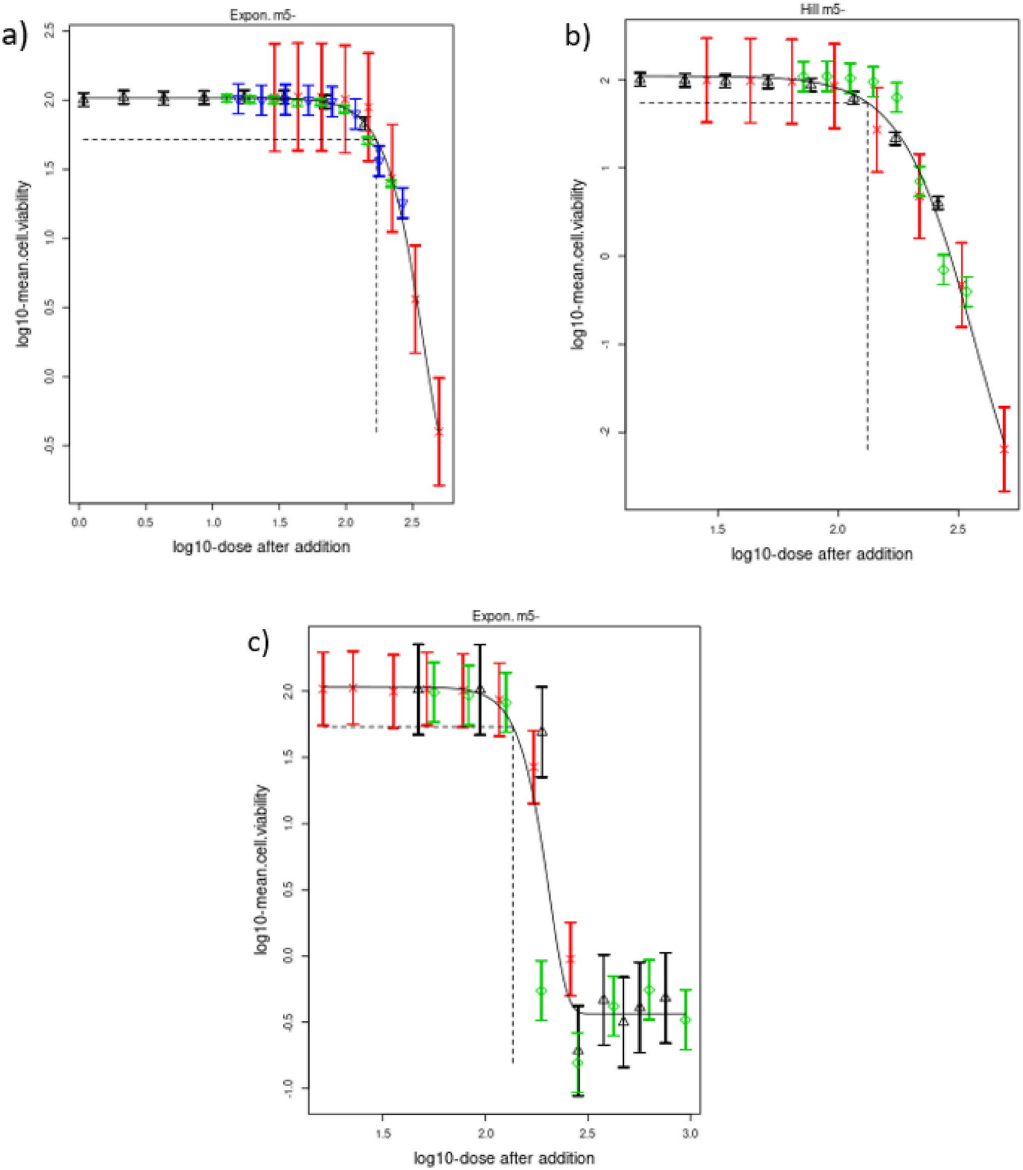
Concentration–response modelling of the cytotoxicity results on HepaRG cells of **a** BP (black upward triangle), NOP (red cross), Pro (green diamond), combination of co-formulants and Pro (dark blue downward triangle), **b** Pen+Fluf (black upward triangle), SN (green diamond), combination of active substances and SN (red cross), **c** Teb + Pro (red cross), NDA (black upward triangle), the combination of active substances and NDA (green diamond). The black lines are representing the dose–response curves of the mixtures under the assumption of dose addition of the individual treatments. Modelling was performed using PROAST software ver.70.1 (colour figure online)

All toxicity data referring to Products 4, 5 and 6 were subsequently subjected to concentration addition modelling in order to confirm any mixture effects observed, that is, concentration addition or over-addition (Fig. 7). In the matter of Product 4, the mixture of Pro + NOP + BP fitted well on the modelled effect curve, indicating that the mixture follows a concentration addition behaviour (Fig. 7a). Similarly, for Product 5, Pen + Fluf + SN was in line with the modelled response curve, showing an additive effect (Fig. 7b). In contrast, for Product 6, the active substances combination with NDA fitted on the modelled response curve for most of the concentrations. However, cytotoxic concentrations indicated a more than additive effect (Fig. 7c).

### Pgp ATPase assay

**Fig. 8:**
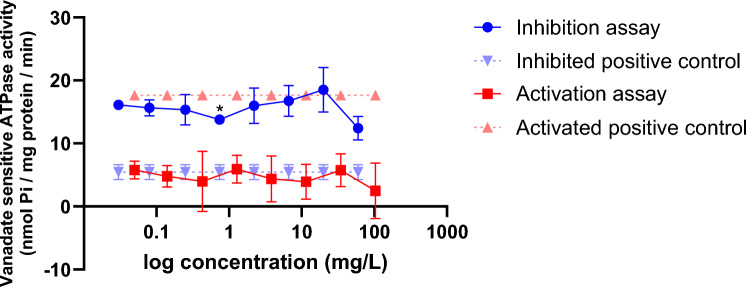
Vanadate sensitive ATPase activity (nmol Pi/mg protein/min) in MDR1-Sf9 membrane vesicles exposed to 0.05–103 mg/L Pro (Activation assay) and 0.03–59.8 mg/L NDA (Inhibition assay). Verapamil (18.2 mg/L) was used as the positive control for the activation assay, whereas cyclosporin (48.1 mg/L) was used as the positive control for the inhibition assay. Results are presented as mean ±SD from 2 independent experiments performed in duplicates. Statistical analysis was done by a linear mixed-effects ANOVA (α = 0.05) followed by a post hoc Dunnett test (*α* = 0.05). Statistical significance compared to the untreated control is indicated by an asterisk (*)

The substrate interaction of Pro and the inhibitory interaction of NDA with the transporter P-gp was performed using a P-gp-specific ATPase assay where the liberation of P_i_ was measured in the presence of both Pro and NDA. In the activation assay, increasing concentration of Pro from 0.05–103.29 mg/L did not stimulate ATPase activity compared to the activated positive control confirming that there is no substrate relationship between Pro and P-gp at all concentrations tested. Concerning the inhibition assay, there was no effect of NDA on the liberation of P_i_ compared to the inhibited positive control demonstrating that NDA does not inhibit P-gp ATPase activity at the selected concentration range (0.03–59.8 mg/L) (Fig. 8). The assay could not be performed with concentrations beyond 300 μM due to the detection range limit of the assay.

### P450-Glo™ CYP2C19 and CYP1A2 inhibition assay

**Fig. 9:**
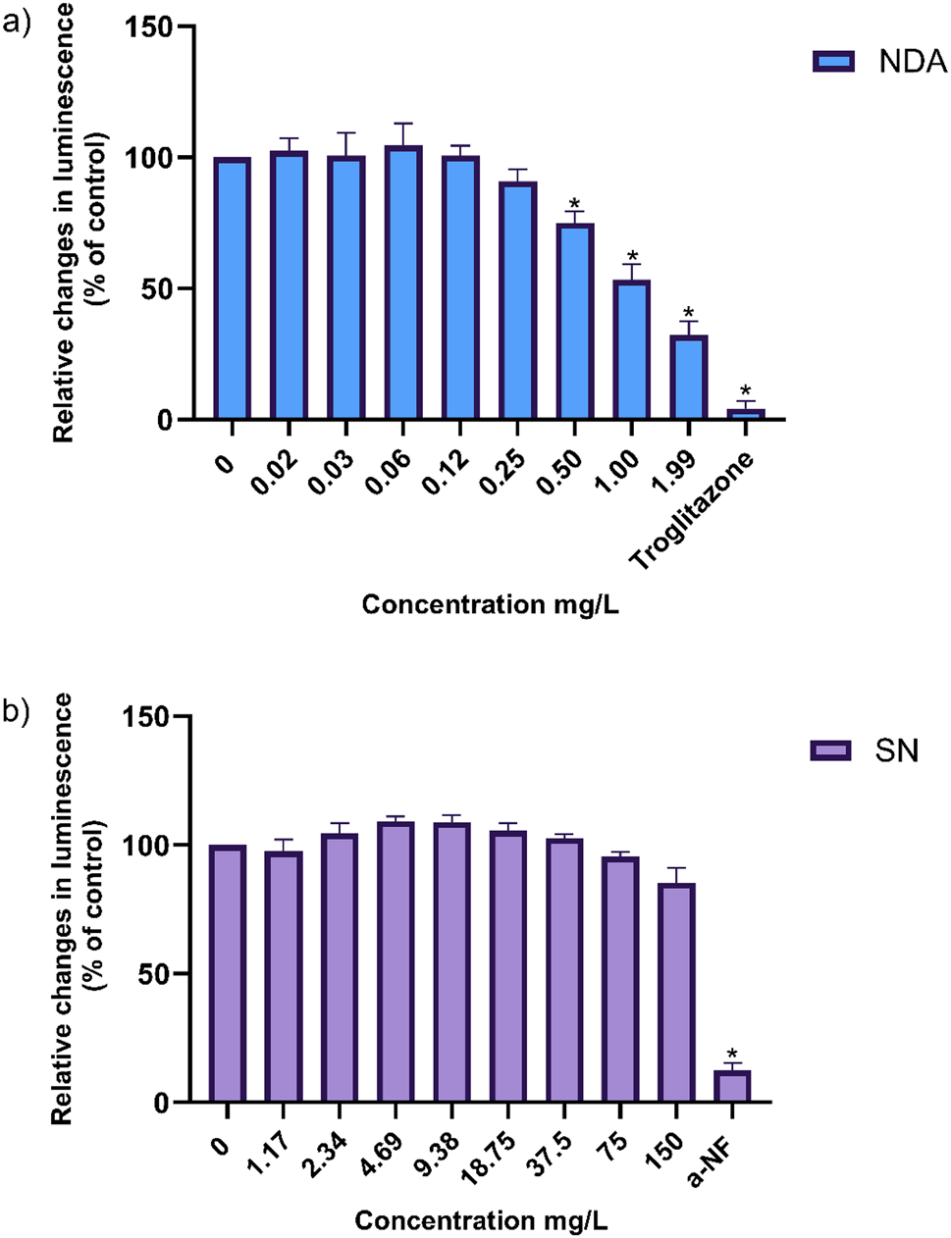
**a** Activity of CYP2C19 is presented as relative changes in luminescence compared to the control following increasing concentration of NDA in mg/L. IC_50_ value calculation was performed using GraphPad Prism software and a four parameters model: *Y* = Bottom + (Top-Bottom)/(1 + 10^((LogIC50 − X)*HillSlope)). **b** Activity of CYP1A2 is presented as relative changes in luminescence compared to the control following increasing concentration of SN in mg/L. Troglitazone (4.42 mg/L) was used as the positive control for CYP2C19 activity, whereas α-naphthoflavone (a-NF) (0.272 mg/L) was used as the positive control for the CYP1A2 activity. Mean values ± SD from three independent replicates, each performed in three technical replicates. Statistical analysis was done by a linear mixed-effects ANOVA (*α* = 0.05) followed by a post hoc Dunnett test. Statistical significance compared to solvent control is indicated by an asterisk (*)

P450-Glo^TM^ assay kits are able to investigate the effects of test compounds on the enzyme activity of a CYP of interest. The P450-Glo™ CYP2C19 and CYP1A2 inhibition assays were used to assess CYP2C19 and CYP1A2 inhibition, respectively, as an underlying cause for any of the observed mixture effects. NDA inhibited CYP2C19 activity in a concentration-dependent manner starting with statistically significant changes at concentrations of 0.5, 1 and 1.99 mg/L. In addition, an IC_50_ value of 0.808 mg/L was derived for NDA (Fig. 9a). On the contrary, SN did not exhibit any inhibition of the CYP1A2 enzyme activity up to the highest concentration tested, 150 mg/L (Fig. 9b).

## Discussion

We applied a NAM-based strategy to prioritise PPP for non-dietary risk assessment, where AS-based assessment might not be sufficient to account for mixture effects. Using in silico predictions for hepatotoxicity, nephrotoxicity and kinetic interactions, we prioritised co-formulants and the PPPs they contain for further in vitro testing. Out of six PPPs that were tested for hepatotoxicity in HepaRG cells, five were more toxic than their corresponding AS and one indicated more than additive effects (Fig. 7c). Here, we discuss (i) whether this NAM-based strategy is useful to prioritise PPPs for mixture-based risk assessment and (ii) whether the current toxicodynamic-based mixture risk assessment strategy needs to be amended to include toxicokinetic interactions.

The hepatotoxicity and nephrotoxicity of co-formulants was investigated by the QSAR tools Leadscope Model Applier (statistical model) and Derek Nexus (expert-based model). With regard to hepatotoxicity, 36 co-formulants were flagged in 161 PPPs, whilst 31 co-formulants were flagged in 235 PPPs with respect to nephrotoxicity endpoint (Fig. 4a, b). The use of specific target organ in silico predictions resulted in a total unique number of 285 prioritised PPPs for further testing out of the 1603 currently authorised in Germany. In addition, making use of ADMET predictor, a number of 56 different co-formulants were flagged with potential kinetic interactions between AS in 215 PPPs (Fig. 5). The addition of in silico ADME predictions step increased the number of PPPs prioritised due to potential kinetic interactions between AS and co-formulants and therefore, minimising the chances of overlooking such interactions contributing to PPP toxicity. Overall, the implementation of both specific target organ and ADME in silico predictions reduced the selection of PPPs for further in vitro testing from 1603 to 427.

It is important to indicate that co-formulants with attributed SMILES were taken into account in this prioritisation scheme, and yet, the remaining ones frequently fall under the group of polymers, minerals and natural extracts could not be considered. In general, SMILES codes may not cover all chemical groups (Benfenati 2022). Therefore, the total sales number of co-formulants was taken into account for prioritising PPPs. Notably, NDA and SN appeared to be the 3^rd^ and 4^th^ best-selling co-formulants (Table 1), respectively, and therefore, one PPP each containing the aforementioned co-formulants was considered for further in vitro cytotoxicity testing. In addition to in silico prediction, this extra step of prioritising PPPs based on co-formulants sales was added since the level of real-life exposure is neglected when only in silico *tools* are used. Where high exposure is linked to substances or extracts of toxicological concern, co-formulant-based risk assessment may be warranted, and data gaps should be closed using appropriate in vitro approaches. Yet, most of the co-formulants registered under REACH are of high production volume and thus, thoroughly assessed for different endpoints (EFSA 2024). Nevertheless, high exposure co-formulants should be investigated in vitro for active transport and metabolic enzyme inhibition. Since toxicokinetic interactions are associated with the concentration investigated, approaches are needed to consider concentration-dependent effects due to co-formulants in PPP risk assessment. For SN, the hypothesised CYP1A2 inhibition could not be confirmed in vitro (Fig. 9b). According to a study by Shimada and Guengerich (2006), not all PAHs have the capacity to inhibit CYP1A2.

Where in silico tools can be applied, it is recommended to use two complementary tools in order to increase the sensitivity and reliability of the predictions (JRC 2010; Worth et al. 2011). QSAR predictivity is generally poor in the case of a complex endpoint such as hepatotoxicity where the structure–activity relationship is less straightforward due to multiple mechanisms of action (Low et al. 2011). Due to the high sensitivity of the hepatotoxic statistical models (i.e. approx. 75%), co-formulants with scores of 2 and 1 were further taken into consideration for in vitro testing, whereas the scores of 2 and 1 for nephrotoxic co-formulants were not considered due to the low sensitivity of the training dataset (i.e. approx. 50%). The concept of the proposed scoring aims to ideally identify more true-positive results and rather true-negative predictions. However, whilst the hepatotoxic models reported in this study are applicable to drugs and based on human data, it is not clear to what extent they are applicable to other types of chemicals. Hepatotoxic models have different abilities to predict hepatotoxicity within a defined region of chemical space, e.g. cholestasis or bile duct disorder. Regardless, such a complex endpoint is unlikely to be predicted by a single model (Ellison et al. 2022). Few (Q)SAR studies of nephrotoxicity have been published, and those identified focus on distinct and small groups of compounds, which limits the applicability of the resulting models (Lapenna et al. 2010).

In addition to organ toxicity, kinetic interaction involving co-formulants was investigated. Product 6 was prioritised in silico for potential Pro, Teb and NDA interactions. Both AS are included in the same CAG by EFSA based on the induction of hepatic toxicity in rodent animal studies (Nielsen et al. 2012), whereas NDA, which is a solvent, is classified as “harmful to aquatic life with long lasting effects, causes serious eye irritation, skin irritation and may cause respiratory irritation” (ECHA 2024). Despite the fact that Pro was flagged as a potential substrate of P-gp active transporter according to ADMET Predictor, the in vitro follow-up showed no interaction with the respective active transporter. Concomitantly, NDA did not elicit any inhibitory effect on the P-gp active transporter in vitro (Fig. 8), whilst ADME predictions indicated the opposite. The binary “yes” or “no” outcome provided by an in silico prediction may not be relevant to the concentrations used in in vitro tests where actual responses depend on factors such as solubility, diffusion into cells, and interaction with cellular components. In addition, these discrepancies may be attributed to ADMET predictor’s training dataset which mainly consists of drug data. Despite the fact that in silico ADME tools using machine learning (ML) models have demonstrated superior predictive accuracy for ADME properties compared to traditional (Q)SAR models (Sakiyama 2009; Racz et al. 2021; Gawehn et al. 2024), the accuracy of ADME predictions of an in silico tool depends on the quality of the experimental data under the database but also the applicability domain (Kar and Leszczynski 2020). Discrepancies between in silico predictions and experimental values do occur, but this does not imply that in silico tools cannot provide valuable assistance for risk assessment (Eleftheriadou et al. 2019). Besides, modelling P-gp–substrate interactions effectively is extremely difficult since P-gp is highly promiscuous due to its ability to undergo significant conformational changes upon binding with different ligands (Chen et al. 2018). In addition to P-gp, more active transporter models are urgently required to cover all relevant uptake and elimination processes in the human body.

Furthermore, Teb was flagged as a potential CYP2C19 substrate, whereas NDA was flagged as a potential inhibitor. In vitro testing confirmed NDA’s CYP2C19 inhibitory potential with an IC_50_ value of 0.808 mg/L (Fig. 9a). A review study on the comparison of different ADME tools with respect to metabolism has shown that ADMET Predictor has the highest sensitivity for identifying inhibitors but the poorest specificity across all software applications used (Zhai et al. 2023). In terms of metabolism, 15% of the pesticides are metabolised by CYP2C19, as well as other CYPs such as CYP3A4 and CYP1A2. In particular, CYP2C19 is responsible for the desulfurisation of pesticides such as parathion and diazinon (Abass et al. 2012). Furthermore, CYP2C19 can metabolise other triazole fungicides including myclobutanil (Fonseca et al. 2019) and Pro (Perovani et al. 2022). Particularly, CYP2C19 accounts for the whole desulfurization pathway of the Pro phase I metabolism in humans (Perovani et al. 2022). However, Teb is predominantly metabolised to TEBOH by CYP3A4 and CYP2C9 and to a lesser extent by CYP2C19 (Habenschus et al. 2019).

Following in silico and sales based prioritisation, cytotoxicity testing in HepaRG cells was applied as an additional lower tier prioritisation step. We aimed to compare AS and PPP toxicities and select PPPs dominating AS toxicity for further in vitro testing. This is in line with the tiered test strategy proposed by Bloch et al. (2020) to address mixture toxicity in PPPs. The selection of the HepaRG cell line was due to its relevance to the target site and sensitivity. Moreover, HepaRG cells are regarded as a reliable surrogate to human hepatocytes for drug metabolism and toxicity studies (Aninat et al. 2005). In all cases, PPPs were more cytotoxic compared to the individual or combination of AS, except Product 1. This is in line with other studies pointing out that PPPs are more cytotoxic compared to their active ingredients (Ferguson et al. 2022; Adler-Flindt and Martin 2019; Mesnage et al. 2014). In fact, various studies highlight the influence of adjuvants such as surfactants and solvents in the increased toxicity of active ingredients of pesticides (Mesnage and Antoniou 2018; Kaisarevic et al. 2019; Karaca et al. 2023). Yet, high tiered testing is required since false positive results may arise from the cytotoxicity assays due to inadequate consideration of the substances’ uptake across biological barriers or their distribution within tissues. The dermal route is of chief importance for operator exposure and provides a barrier for AS and co-formulant absorption. Substance concentrations will be greatly reduced and relative PPP composition will be changed following this step. Therefore, this prioritisation strategy provides a worst-case scenario and is aimed at identifying PPPs with the potential for co-formulant-based or synergistic effects. Prioritised PPPs require further testing. Moreover, additional NAM-based models should be applied as cytotoxicity in liver cells may not always be a reliable indicator endpoint. Thus, the combined use of different living systems such as HepaRG cells and zebrafish embryos as a first tiered test approach to investigate potential acute and sub-chronic toxicities, respectively, is recommended (Bloch et al. 2020).

Comparative cytotoxicity combined with the investigation of kinetic interaction may, however, provide insights into the mechanisms of mixture toxicity and its effect size. Many studies have reported metabolic induction or inhibition as the cause of mixture toxicity for pesticides and PPPs (Cedergreen 2014; Hernández et al. 2017; Martin et al. 2021). Such interactions are the likely cause of more than additive effects of Teb, Pro and NDA as observed in Product 6 (Fig. 9a). The mixture of Teb + Pro + NDA resulted in a calculated EC_50_ threefold lower compared to the EC_50_ derived from the mixture of the two AS. Concentration addition modelling is commonly used to characterise mixture effects by identifying synergistic or antagonistic interactions of chemical mixtures when observations deviate from expected additivity (Spurgeon et al. 2010; Cedergreen 2014). Such a deviation towards synergistic effects was observed for Product 6 (Fig. 7c). Although synergistic effects are less likely to occur at doses corresponding to dietary exposure to mixtures of pesticide residues (EFSA 2017), they may be relevant for operators mixing and loading and applying PPPs. Ultimately, their relevance is potentially underestimated since effective concentrations of kinetic interaction are not considered when threshold values are derived.

In conclusion, our findings suggest that the consideration of toxicokinetic mixture effects is important to prioritise PPPs. The implementation of in silico ADME predictions in the prioritisation step increased the number of prioritised PPPs for further in vitro testing without neglecting such interactions between hepatotoxic AS and co-formulants and ultimately reduced the overall number of PPPs for further testing. In particular, Product 6 was prioritised based on the in silico kinetic interactions. In addition, it was the only PPP deviating from dose addition modelling, and the calculated EC_50_ value of the mixture of AS with the co-formulant was 3 times lower compared to the mixture of two AS. Therefore, the inclusion of kinetic interactions between AS and co-formulants for the prioritisation of PPPs is recommended. Yet, Product 6 should be considered as a worst-case scenario in this prioritisation scheme. As a follow-up, all 427 prioritised PPPs should be followed up in vitro with an emphasis on the investigation of potential kinetic interactions between AS and co-formulants. This recommendation is supported by De Jong et al. (2022) who identified toxicokinetic influences of co-formulants on pesticide AS as one of the high priority scientific gaps in mixture risk assessment. Braeuning et al. (2022) proposed grouping substances into Common Kinetic Groups (CKGs) based on their ability to induce or inhibit metabolic enzymes and active transporters.

Based on our prioritisation scheme and the limited number of comparative in vitro tests conducted, we extrapolate that higher tier toxicity testing to account for more than additive effects would be required for approximately 100 of the 1600 authorised PPPs considered in this study. Taking into account the aforementioned limitations, this number is an estimate based on this proposed prioritisation scheme. In future, additional endpoints should be addressed by in silico screening, and in vitro-based prioritisation should be extended by small organism testing, e.g. in zebrafish embryos or *C. elegans*. At the same time, we note that the observed deviation from additivity for the relevant compounds in Product 6 was not extensive. In conclusion, our testing strategy effectively reduces the number of PPPs for which extensive mixture testing is required whilst focussing on those products where mixture effects are most likely to occur. Our results indicate that the identification of relevant co-formulants and the subsequent application of the additivity principle could be sufficient for PPP risk assessment. Where synergism is observed, we propose the derivation of points-of-departure (PoDs) from multiple lines of evidence including omics and mechanism-specific in vitro testing. These PoDs form the basis for in vitro–in vivo extrapolation to derive regulatory threshold values for human health risk assessment.

## Supplementary Information

Below is the link to the electronic supplementary material.

Supplementary file1 (DOCX 15 KB)

Supplementary file2 (XLSX 83 KB)

Supplementary file3 (XLSX 47 KB)

Supplementary file4 (XLSX 59 KB)

Supplementary file5 (XLSX 73 KB)

Supplementary file6 (DOCX 581 KB)

## Data Availability

The data that support the findings of this study are available within the paper and its Supplementary Information. Data on the composition of the PPP is subject to confidentiality and cannot be disclosed.

## Abbreviations

ADME: Absorption, distribution, metabolism, excretion
AIC: Akaike information criteria
AS: Active substances
BP: 1-Butylpyrrolidin-2-one
CA: Dose or concentration addition
CAGs: Cumulative assessment groups
Clo: Cloquintocet-mexyl
Cyp: Cypermethrin
Del: Deltamethrin
DMSO: Dimethyl sulfoxide
FCS: Foetal calf serum
Fluf: Flufenacet
(Q)SARs: Quantitative structure–activity relationships
MDR1: Multidrug resistance protein 1
ML: Machine learning
NAMs: New approach methodologies
NDA: N,N-dimethyldecanamide
NOP: 1-Octyl-2-pyrrolidone
NRU: Neutral red uptake
PBS: Phosphate-buffered saline
P-gp: P-glycoprotein
Pin: Pinoxaden
PPPs: Plant protection products
Pro: Prothioconazole
SMILES: Simplified molecular-input line-entry systems
SN: Solvent naphtha
Teb: Tebuconazole
TP: Tris-(2-ethylhexyl)-phosphate
WST-1: Water-soluble tetrazolium
