# Supplementary material for: Prioritisation of co-formulants and plant protection products for non-dietary risk assessment using NAMs
Source: Arch Toxicol. 2025 Jul 2;99(8):3205–21. doi: 10.1007/s00204-025-04078-0 (PMC12367827; doi:10.1007/s00204-025-04078-0)
Supplement: Supplementary file 1 — Supplementary file1 (DOCX 15 KB) [file 204_2025_4078_MOESM1_ESM.docx]

Article name: Prioritisation of co-formulants and plant protection products for risk assessment using NAMs.

Journal name: Archives of Toxicology

Author names: Alkiviadis Stagkos-Georgiadis^1,2^, Bright Baffour-Duah^1,3^, Tewes Tralau^1^ and Denise Bloch^1^

Affiliation: ^1^ Department of Pesticides Safety, German Federal Institute for Risk Assessment (BfR), Max-Dohrn-Str. 8-10, 10589 Berlin, Germany

^2^ University of Potsdam, Institute of Nutritional Science, Department of Nutritional Toxicology, Arthur-Scheunert-Alle 114-116, 14558 Nuthetal, Germany

^3^University of Potsdam, Institute of Nutritional Science, Department of Food Chemistry, Arthur-Scheunert-Alle 114-116, 14558 Nuthetal, Germany

Email address of corresponding author: Corresponding authors: [Alkiviadis.Stagkos-Georgiadis@bfr.bund.de](mailto:Alkiviadis.Stagkos-Georgiadis@bfr.bund.de)

**Table 1S** Training dataset, sensitivity and specificity of hepatotoxic models

| **Hepatotoxic Models** | **Training Compounds** | **Sensitivity**  **(%)** | **Specificity**  **(%)** |
| --- | --- | --- | --- |
| Bile Duct Disorders | 1017 | 75.9 | 86.6 |
| Cholestasis | 1124 | 74.8 | 76.9 |
| Liver Acute Damage | 1314 | 73.2 | 66.1 |
| Liver Enzyme Release Disorders | 1134 | 72.6 | 76.1 |

**Table 2S** Training dataset, sensitivity and specificity of nephrotoxic models

| **Nephrotoxic Models** | **Training Compounds** | **Sensitivity (%)** | **Specificity (%)** |
| --- | --- | --- | --- |
| Bladder Disorders | 1591 | 51.5 | 89.7 |
| Blood in Urine Disorders | 1591 | 49.7 | 94.3 |
| Kidney Disorders | 1590 | 36.8 | 95.8 |
| Kidney Function tests | 1589 | 48.9 | 89.9 |
| Nephropathy Disorders | 1590 | 52.9 | 90.8 |
| Urolithiasis Disorders | 1591 | 42.1 | 94.9 |

**Reference**

Leadscope Inc. An Instem Company 1393 Dublin Road Columbus, OH 43215 Proprietary and Confidential Copyright 2021 by Leadscope, Inc. Version 3.1 September 2021. Open access <https://www.leadscope.com/ls-manuals/>
