# Supplementary material for: Prioritisation of co-formulants and plant protection products for non-dietary risk assessment using NAMs
Source: Arch Toxicol. 2025 Jul 2;99(8):3205–21. doi: 10.1007/s00204-025-04078-0 (PMC12367827; doi:10.1007/s00204-025-04078-0)
Supplement: Supplementary file 6 — Supplementary file6 (DOCX 581 KB) [file 204_2025_4078_MOESM6_ESM.docx]

Article name: Prioritisation of co-formulants and plant protection products for risk assessment using NAMs.

Journal name: Archives of Toxicology

Author names: Alkiviadis Stagkos-Georgiadis^1,2^, Bright Baffour-Duah^1,3^, Tewes Tralau^1^ and Denise Bloch^1^

Affiliation: ^1^ Department of Pesticides Safety, German Federal Institute for Risk Assessment (BfR), Max-Dohrn-Str. 8-10, 10589 Berlin, Germany

^2^ University of Potsdam, Institute of Nutritional Science, Department of Nutritional Toxicology, Arthur-Scheunert-Alle 114-116, 14558 Nuthetal, Germany

^3^University of Potsdam, Institute of Nutritional Science, Department of Food Chemistry, Arthur-Scheunert-Alle 114-116, 14558 Nuthetal, Germany

Email address of corresponding author: Corresponding authors: [Alkiviadis.Stagkos-Georgiadis@bfr.bund.de](mailto:Alkiviadis.Stagkos-Georgiadis@bfr.bund.de)

**Fig. 1** Results of the WST cytotoxicity assay on HepaRG cells after 24h exposure to increasing concentrations of **a)** Product 1 and Cyp as featured in product 1 **b)** Product 2, a combination of Pin+Clo as featured in product 2 and TP **c)** Product 3, a combination of Pip+Del and Pip as featured in product 3, d) Product 4, a combination of Pro+NOP+BP and Pro as featured in product 4, NOP and BP e) Product 5, a combination of Pen+Fluf and a combination of Pep+Fluf+SN as featured in Product 5 and SN f) Product 6, a combination of Teb+Pro and a combination of Teb+Pro+NDA as featured in product 6 and NDA. Mean values SD of n= 3 biological replicates each performed with three technical replicates
